# Attentional Bias Associated with Habitual Self-Stigma in People with Mental Illness

**DOI:** 10.1371/journal.pone.0125545

**Published:** 2015-07-15

**Authors:** Kevin K. S. Chan, Winnie W. S. Mak

**Affiliations:** 1 Department of Psychological Studies, The Hong Kong Institute of Education, Tai Po, Hong Kong; 2 Centre for Psychosocial Health, The Hong Kong Institute of Education, Tai Po, Hong Kong; 3 Department of Psychology, The Chinese University of Hong Kong, Shatin, Hong Kong; Erasmus University Rotterdam, NETHERLANDS

## Abstract

As habitual self-stigma can have a tremendous negative impact on people with mental illness, it is of paramount importance to identify its risk factors. The present study aims to examine the potential contributory role of attentional bias in habitual self-stigma. People with mental illness having strong (*n* = 47) and weak (*n* = 47) habitual self-stigma completed a computerized emotional Stroop task which included stigma-related, positive, and non-affective words as stimuli. The strong habit group was found to exhibit faster color-naming of stigma-related words (compared to non-affective words), whereas the weak habit group showed no difference in the speed of response to different stimuli. These findings suggest that people with stronger habitual self-stigma may be more able to ignore the semantic meaning of stigma-related words and focus on the color-naming task. Moreover, people with stronger habitual self-stigma may have greater attentional avoidance of stigma-related material. The present study is the first to demonstrate a specific relationship between habitual self-stigma and biased processing of stigma-related information. In order to further determine the role and the nature of attentional bias in habitual self-stigma, future research should employ a broader range of experimental paradigms and measurement techniques to examine stigma-related attentional bias in people with mental illness.

## Introduction

The stigma of mental illness is a worldwide concern that adversely impacts the life opportunities and psychological well-being of many people with mental illness. The affected individuals may experience public stigma (i.e., the prejudice and discrimination that result from the general population endorsing stereotypes about mental illness) as well as self-stigma (i.e., the harm to self-esteem that results from internalizing cultural stereotypes about mental illness) [1]. Self-stigma, which is the focus of the present study, has been found to be associated with negative outcomes for people with mental illness. Specifically, self-stigma is related to lowered self-efficacy [2], empowerment [3], morale [4], quality of life [5], and treatment adherence [6]. Self-stigma is also related to an increase in depressive symptoms [7] and social anxiety symptoms [8].

To date, theories of self-stigma have been focused on explaining why some people with mental illness internalize cultural stereotypes of mental illness (e.g., “People with mental illness are dangerous and socially incompetent.”) and form negative self-perceptions (e.g., “Because I have a mental illness, I am dangerous and socially incompetent.”). Link [9] proposed that cultural stereotypes of mental illness may take on personal relevance among individuals who accept the diagnostic label of “mental illness”. Corrigan [10] suggested that people with mental illness who are aware of and agree with these stereotypes may apply negative and stigmatizing views to themselves. Such self-stigmatization undermines the individuals’ mental health and life satisfaction, and acts as a barrier to their help-seeking and recovery [11–14].

Albeit valuable, existing theories of self-stigma are focused largely on elucidating why it is initially acquired, with little discussion on how it is sustained and perpetuated across time. However, in fact, while many people with mental illness may self-concur with the “content” of stigmatizing thoughts at some point, they may have different degrees of habitual recurrence of such thoughts, which could exacerbate the experience of self-stigma and perpetuate its adverse effects on mental health and recovery [15–17]. Although it is important to understand the “process” of how self-stigmatizing thoughts unfold in the everyday lives of people with mental illness, to date, only limited research has been conducted to conceptualize and distinguish the habitual “process” of self-stigma from its cognitive “content” [18].

Chan and Mak [18,19] recently clarified the relationship between the content and the process of self-stigma based on a mental habit research paradigm, which has been used to illustrate the content-process distinction in negative self-thinking [20], negative body perception [21], worrying [22], and narcissism [23]. Under this paradigm, the content of self-stigma refers to the extent of self-concurrence with negative stereotypes about mental illness (*stereotype self-concurrence*), whereas its process refers to the extent to which these self-stigmatizing thoughts emerge habitually in everyday life (*habitual self-stigma*). It is expected that the cognitive content of self-stigma by itself can dampen one’s well-being [13]; however, several intrapersonal characteristics (e.g., ruminative coping with self-stigma) and social-contextual factors (e.g., exposure to public discrimination) may further exacerbate the adverse effects of self-stigma by leading to the frequent activation of self-stigmatizing thoughts. When self-stigmatizing thinking occurs repetitively and persistently, and becomes a dominant feature of the mind, it may eventually develop into a mental habit [19].

A mental habit refers to a thought that has acquired a certain degree of automaticity after repetition [20]. Likewise, a mental habit of self-stigma (a.k.a. habitual self-stigma) is characterized by the repeated and automatic occurrence of self-stigmatizing thought. To date, studies have already shown that habitual self-stigma significantly predicts lowered self-esteem, life satisfaction, and personal recovery, even after controlling for the effects of stereotype self-concurrence [18,24]. Given the significant adverse effects of habitual self-stigma on psychological outcomes, it is of paramount importance to identify its risk factors and to understand the underlying psychological mechanisms of its development.

Although research on habitual self-stigma has only recently attracted interest, habitual negative thinking and its risk factors have long been studied in the field of affective disorders. Some authors pointed out that depressive and anxiety disorders are characterized by habits of negative thoughts and by difficulties in overriding these habits [25,26]. Cognitive models of psychopathology posit that these mental habits are characterized by information-processing biases (e.g., attentional bias), with the cognitive content of these biases being mood-congruent and disorder-specific [27]. For instance, depression is related to attentional bias towards stimuli conveying sadness, loss, or hopelessness, whereas anxiety is related to attentional bias for threat-related stimuli [27]. These attentional biases are thought to explain the development and maintenance of habitual negative thoughts in depression and anxiety [27]. With these prominent cognitive models of mental habits, the role of attentional bias in habitual self-stigma is potentially worthy of investigation. A relevant research question is whether people with stronger habitual self-stigma tend to show greater attentional bias towards stigma-related information. By means of such attentional bias, the individuals’ stigmatized identity may become highly salient and emerge more frequently on their minds. Their self-stigmatizing thoughts may then be activated more often, potentially causing habitual self-stigma.

One task that has been commonly used to assess attentional bias for specific stimuli is the emotional Stroop task [28]. This task is a variant of the cognitive Stroop paradigm, which is a prominent test of selective attention [29,30]. In the cognitive Stroop task, words of color names are printed in colors different from the meaning of the words; participants are required to name the ink color of the words (rather than performing the automatic and overlearned response of word-reading). The color identification process is interfered if attention is drawn to the semantic meaning of the words. Such attentional interference gives rise to prolonged response latencies, reflecting the failure to focus attention on the task-relevant feature of the stimuli. In a similar vein, the emotional Stroop task utilizes written stimuli with an emotional valence to interfere with attentional processes. Prolonged latencies for color-naming affective words (compared to non-affective words) are thought to indicate attentional interference by the emotional characteristics of the affective stimuli. To date, the emotional Stroop paradigm has been widely adapted to assess attentional biases in various affective disorders that involve habitual negative thoughts [31,32]. Research has shown that many people with depressive and anxiety disorders exhibit increased latencies to color-name emotionally negative words (e.g., depression- and threat-related words) compared to positive or non-affective words, indicating their attentional bias towards mood-congruent information [31,32]. However, there is no evidence thus far for attentional bias in habitual self-stigma.

The present study aims to examine how habitual self-stigma is related to stigma-related attentional bias in people with mental illness. To achieve this research goal, we compared the performance of people with strong and weak habitual self-stigma on an emotional Stroop task. We hypothesized that the strong habit group would show greater attentional bias towards stigma-related stimuli, which would be reflected through prolonged latencies for color-naming stigma-related words (compared to non-affective words). To rule out the possibility that any observed emotional Stroop effects of stigma-related stimuli were due to the participants’ stereotype self-concurrence and depressive symptoms (which are known correlates of habitual self-stigma and possibly associated with an attentional bias for negatively valenced material), we measured these two constructs as control variables. Additionally, we measured the participants’ attentional bias for positively valenced material in order to rule out the possibility that any observed emotional Stroop effects of stigma-related stimuli were explained by the affective nature of the stimuli rather than their stigmatizing valence per se. Finally, the study included a cognitive Stroop task in order to rule out the possibility that any observed emotional Stroop effects were simply a function of the general selective attention capacity.

## Methods

### Ethics Statement

The present study was approved by the Clinical Research Ethics Committee of The Chinese University of Hong Kong. Written informed consent was attained from all participants prior to participation.

### Participants

Participants were recruited through referral from four community mental health centers in Hong Kong. Inclusion criteria were a diagnosis of a DSM-IV-TR Axis I disorder and Cantonese-speaking Chinese. Exclusion criteria were a diagnosis of intellectual disability or color blindness and a history of major medical or neurological illness. Diagnostic information was obtained from medical records.

A total of 161 consecutive people with mental illness were screened and completed a measure of habitual self-stigma, the Self-stigmatizing Thinking’s Automaticity and Repetition (STAR) scale [24]. In order to identify the participants who had strong and weak habitual self-stigma, we used a quartile split method to pinpoint high and low scorers on the STAR. Only the participants who scored in the top or bottom quartile were recruited for the present study. Those who scored in the top quartile were classified as having strong habitual self-stigma, whereas those who scored in the bottom quartile were classified as having weak habitual self-stigma.

### Measures

#### Habitual self-stigma

To assess the habitual process of self-stigma, participants completed the 8-item STAR scale. The STAR was adapted from a widely used measure of mental habit, the Habit Index of Negative Thinking (HINT) [20]. The HINT was originally developed for nonclinical populations to assess habitual negative self-thinking and has been adapted to measure different mental habits [21–23]. To develop the STAR, we changed the introductory clause for the HINT items to emphasize habitual self-stigma. That is, the clause “Thinking negatively about myself is something” was modified to “Thinking negatively about my identity as a person with mental illness is something”. Subsequent clauses were adapted from the HINT. Each of these clauses taps onto one feature of habitual self-stigma: repetitive (e.g., “I do frequently.”) and automatic (e.g., “I do without further thinking.”). Participants were required to rate each item on a 5-point Likert scale from (1) *strongly disagree* to (5) *strongly agree*. Higher scores indicated a greater level of habitual self-stigma. The STAR has been validated and used to assess habitual self-stigma in people with mental illness [18,19,24]. In the present study, the internal consistency of the STAR was excellent (Cronbach’s alpha = 0.95).

#### Stereotype self-concurrence

To assess the cognitive content of self-stigma, participants completed the 9-item Self-Stigma Scale-Short Form (SSS-S) [33]. Participants were required to rate each item on a 6-point Likert scale from (1) *strongly disagree* to (6) *strongly agree*. Higher scores indicated a greater level of stereotype self-concurrence. In the present study, the internal consistency of the SSS-S was excellent (Cronbach’s alpha = 0.94).

#### Depression

To assess the severity of depressive symptoms, participants completed the 6-item depression/functioning subscale of the Revised Behavior and Symptom Identification Scale (BASIS-R-D/F) [34]. Participants were required to rate each item on a 5-point Likert scale from (0) *never* to (4) *always*. Higher scores indicated a greater severity of depressive symptoms. In the present study, the internal consistency of the BASIS-R-D/F was good (Cronbach’s alpha = 0.89).

#### Attentional bias for stigma-related and positive stimuli

To assess attentional bias for stigma-related and positive stimuli, participants completed an emotional Stroop task [28], which was programmed with E-Prime 2.0 and administered on a laptop computer. This task was designed to measure the latencies to name the ink color of stigma-related words (i.e., failure, stupid, incompetent, weak, lazy, violent) versus positive words (i.e., proud, strong, confident, bold, daring, fearless) versus non-affective words (i.e., flower, skill, urban, immortal, wavering, deciding). The stigma-related words were common adjectives used to describe negative stereotypes about mental illness; the positive words were chosen from the list of self-assurance words in the Positive and Negative Affect Schedule—Expanded Form [35]; the non-affective words were selected from those used in previous studies [36]. These words were grouped into three emotional categories (i.e., stigma-related, positive, non-affective), each of which was presented in a single block of 24 test trials. The sequence of blocks was counterbalanced among participants. The three blocks of words were presented in Chinese and matched for word frequency [37] and complexity. In each block, each of the six words appeared once in red, yellow, blue, and green colors, respectively. Participants had to perform the color-naming task by pressing four marked response keys. Two scores were calculated for each block: (1) the error rate and (2) the mean reaction time of the correct trials. In addition, an attentional bias score was calculated per emotional condition by subtracting the mean reaction time of the non-affective trials from that of the emotional trials. A positive score represented an emotional Stroop interference effect (i.e., greater attentional interference produced by the emotional condition compared to the non-affective condition), whereas a negative score represented an emotional Stroop facilitation effect (i.e., less attentional interference produced by the emotional condition compared to the non-affective condition).

#### Selective attention

To assess the general selective attention capacity, participants completed a cognitive Stroop task [38], which was programmed with E-Prime 2.0 and administered on a laptop computer. In this task, the stimuli consisted of Chinese words of color names (i.e., red, yellow, blue, green). The stimuli were printed in colors different from the meaning of the words. In one block of 24 test trials (a.k.a. the word-naming block), participants were required to identify the semantic meaning of each stimulus. In another block of 24 test trials (a.k.a. the color-naming block), participants were required to identify the ink color of each stimulus. Participants had to give their responses by pressing four marked response keys. Two scores were calculated for each block: (1) the error rate and (2) the mean reaction time of the correct trials. In addition, a selective attention score was calculated by subtracting the mean reaction time of the word-naming trials from that of the color-naming trials. A positive score represented a cognitive Stroop interference effect. Higher scores indicated a lower level of selective attention capacity (i.e., greater difficulty in focusing attention on the task-relevant feature of the stimuli).

### Data Analyses

All statistical analyses were carried out using the Statistical Packages for Social Sciences (SPSS) version 22.0. To examine group differences in the error rates of the emotional and cognitive Stroop trials, two independent samples *t*-tests were carried out. To compare the response latencies in the emotional Stroop task between the two participant groups, a repeated measures analysis of variance (ANOVA) for the latency scores was performed with two between-subject levels (“Group”: the strong and weak habit groups) and three within-subject levels (“Condition”: the stigma-related, positive, and non-affective blocks). To compare the response latencies in the cognitive Stroop task between the two groups, a repeated measures ANOVA for the latency scores was performed with two between-subject levels (“Group”: the strong and weak habit groups) and two within-subject levels (“Condition”: the word-naming and color-naming blocks). To examine group differences in the attentional bias and selective attention scores, a total of three independent samples *t*-tests were carried out.

## Results

### Participant Characteristics

Participant characteristics are presented in Table 1. The two participant groups did not differ significantly in sociodemographic and clinical characteristics. However, the strong habit group had significantly greater levels of habitual self-stigma, stereotype self-concurrence, and depression than the weak habit group. As stereotype self-concurrence and depression, which showed significant group differences, were not significantly correlated with the dependent variables of the study, these two constructs were not included as covariates in the subsequent analyses.

**Table 1:** Characteristics of participants in the strong and weak habit groups.

	Strong habit group (*n* = 47)	Weak habit group (*n* = 47)	Statistics: χ^2^ or *t*	*p*
***Sociodemographic characteristics***				
Male, *n* (%)	25 (53.2)	25 (53.2)	0	1
Age, years, mean (*SD*)	39.09 (11.39)	42.06 (9.76)	-1.36	0.18
Highest educational level			4.98	0.08
Less than high school, *n* (%)	13 (27.6)	19 (40.4)		
High school, *n* (%)	32 (68.1)	22 (46.8)		
College, university, or graduate school, *n* (%)	2 (4.3)	6 (12.8)		
Employment status			2.15	0.34
Employed, *n* (%)	9 (19.1)	14 (29.8)		
Unemployed, *n* (%)	32 (68.1)	30 (63.8)		
Not in labor force (i.e., volunteer, student, retired), *n* (%)	6 (12.8)	3 (6.4)		
Marital status (i.e., married versus single, separated, divorced, or widowed)			0.71	0.40
Married, *n* (%)	9 (19.1)	6 (12.8)		
Single, *n* (%)	33 (70.2)	38 (80.8)		
Separated, *n* (%)	1 (2.1)	1 (2.1)		
Divorced, *n* (%)	2 (4.3)	2 (4.3)		
Widowed, *n* (%)	2 (4.3)	0		
***Clinical characteristics***				
Duration of illness, years, mean (*SD*)	12.66 (8.32)	14.60 (8.23)	-1.14	0.26
Psychiatric diagnosis (i.e., schizophrenia-spectrum disorders versus affective disorders)			0.85	0.36
Schizophrenia, *n* (%)	29 (61.7)	33 (70.2)		
Schizoaffective disorder, *n* (%)	0	1 (2.1)		
Delusional disorder, *n* (%)	2 (4.3)	1 (2.1)		
Psychosis, *n* (%)	1 (2.1)	1 (2.1)		
Bipolar disorder, *n* (%)	4 (8.4)	7 (15)		
Major depressive disorder, *n* (%)	7 (15)	3 (6.4)		
Obsessive-compulsive disorder, *n* (%)	3 (6.4)	0		
Posttraumatic stress disorder, *n* (%)	1 (2.1)	0		
Panic disorder, *n* (%)	0	1 (2.1)		
***Other characteristics***				
STAR, mean (*SD*)	3.97 (0.37)	2.12 (0.41)	23.16	< 0.001
SSS-S, mean (*SD*)	4.20 (1.11)	2.74 (0.94)	6.89	< 0.001
BASIS-R-D/F, mean (*SD*)	1.97 (0.69)	0.75 (0.65)	8.77	< 0.001

STAR = Self-stigmatizing Thinking’s Automaticity and Repetition; SSS-S = Self-Stigma Scale-Short Form; BASIS-R-D/F = Depression/Functioning subscale of the Revised Behavior and Symptom Identification Scale.

### Response Errors in the Emotional Stroop Task

The two groups did not differ significantly in the error rate of the emotional Stroop trials (strong habit = 2.4%, weak habit = 3.2%; *t* = 1.07, *p* = 0.29). The error rate was not significantly correlated with the mean reaction time of the trials (*r* = 0.20, *p* = 0.06). A speed-accuracy trade-off was thus unlikely to be a significant determinant of the participants’ task performance.

### Response Latencies in the Emotional Stroop Task

**Fig 1:**
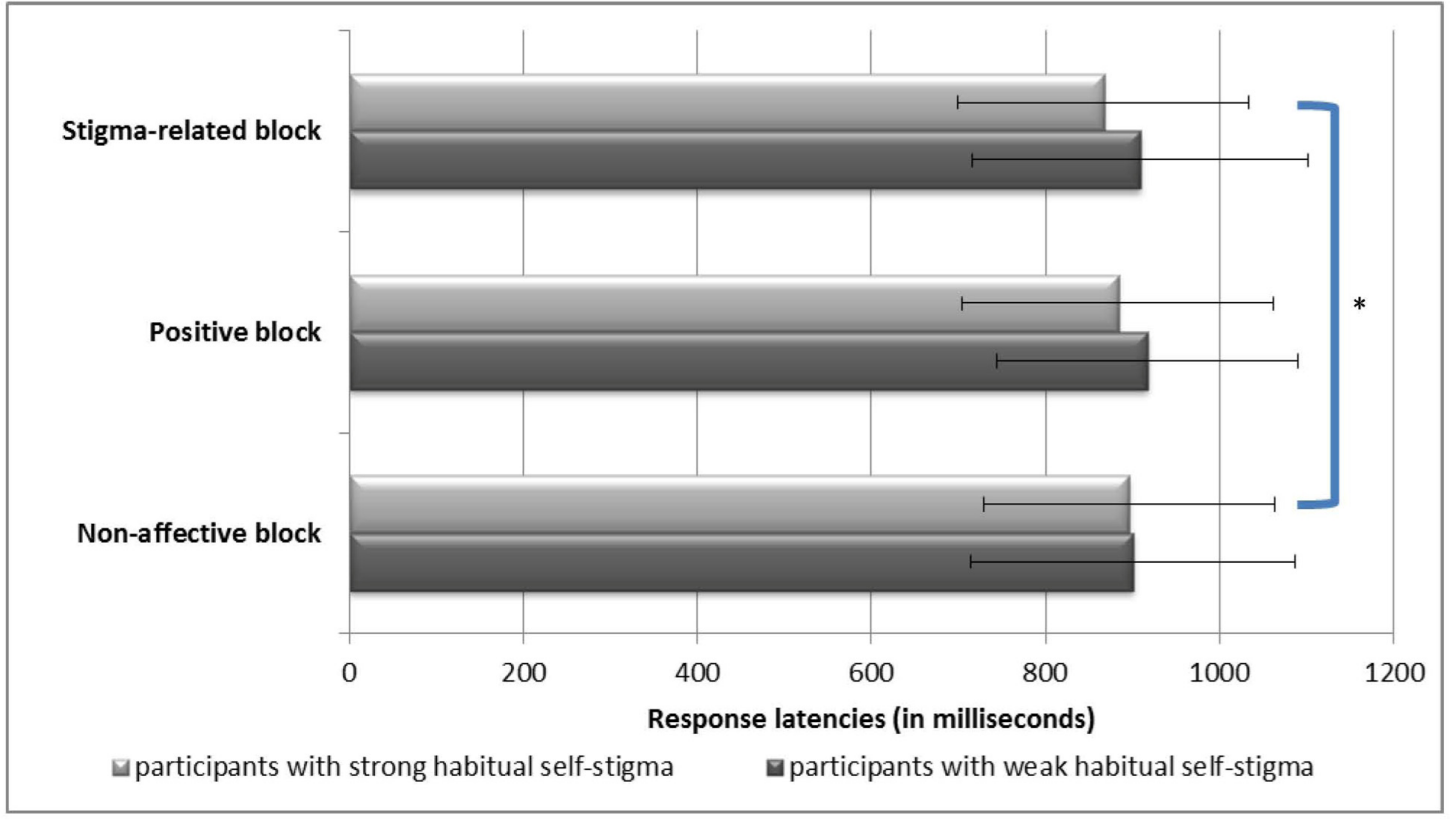
Participants’ performance on the emotional Stroop task. Mean response latencies in the stigma-related, positive, and non-affective blocks. An asterisk indicates a significant difference.

A repeated measures ANOVA for the latency scores showed that the interaction effect of “Group” X “Condition” was significant (*F* = 3.13, *p* = 0.048); however, the main effects of “Group” (*F* = 0.59, *p* = 0.45) and “Condition” (*F* = 1.35, *p* = 0.26) were not significant (see Fig 1). A follow-up paired samples *t*-test showed that the strong habit group had faster reaction times during the stigma-related condition compared to the non-affective condition (*p* = 0.04), whereas the weak habit group did not show such effect.

According to the attentional bias scores calculated for the stigma-related condition, the strong habit group showed an emotional Stroop facilitation effect (mean = -29.52 ms, standard deviation = 79.75 ms), whereas the weak habit group showed an emotional Stroop interference effect (mean = 8.34 ms, standard deviation = 69.37 ms). An independent samples *t*-test showed that the two groups differed significantly in the magnitude of the emotional Stroop effect (*t* = -2.46, *p* = 0.016).

According to the attentional bias scores calculated for the positive condition, the strong habit group showed an emotional Stroop facilitation effect (mean = -12.61 ms, standard deviation = 75.44 ms), whereas the weak habit group showed an emotional Stroop interference effect (mean = 16.43 ms, standard deviation = 100.10 ms). An independent samples *t*-test showed that there was no significant group difference in the magnitude of the emotional Stroop effect (*t* = -1.59, *p* = 0.12).

### Response Errors in the Cognitive Stroop Task

The two groups did not differ significantly in the error rate of the cognitive Stroop trials (strong habit = 10.6%, weak habit = 14.6%; *t* = 1.29, *p* = 0.20). The error rate was positively correlated with the mean reaction time of the trials (*r* = 0.71, *p* < 0.001), indicating that participants who responded slower committed more errors than those responded faster. As such, a speed-accuracy trade-off was unlikely to be a significant determinant of the participants’ task performance.

### Response Latencies in the Cognitive Stroop Task

**Fig 2:**
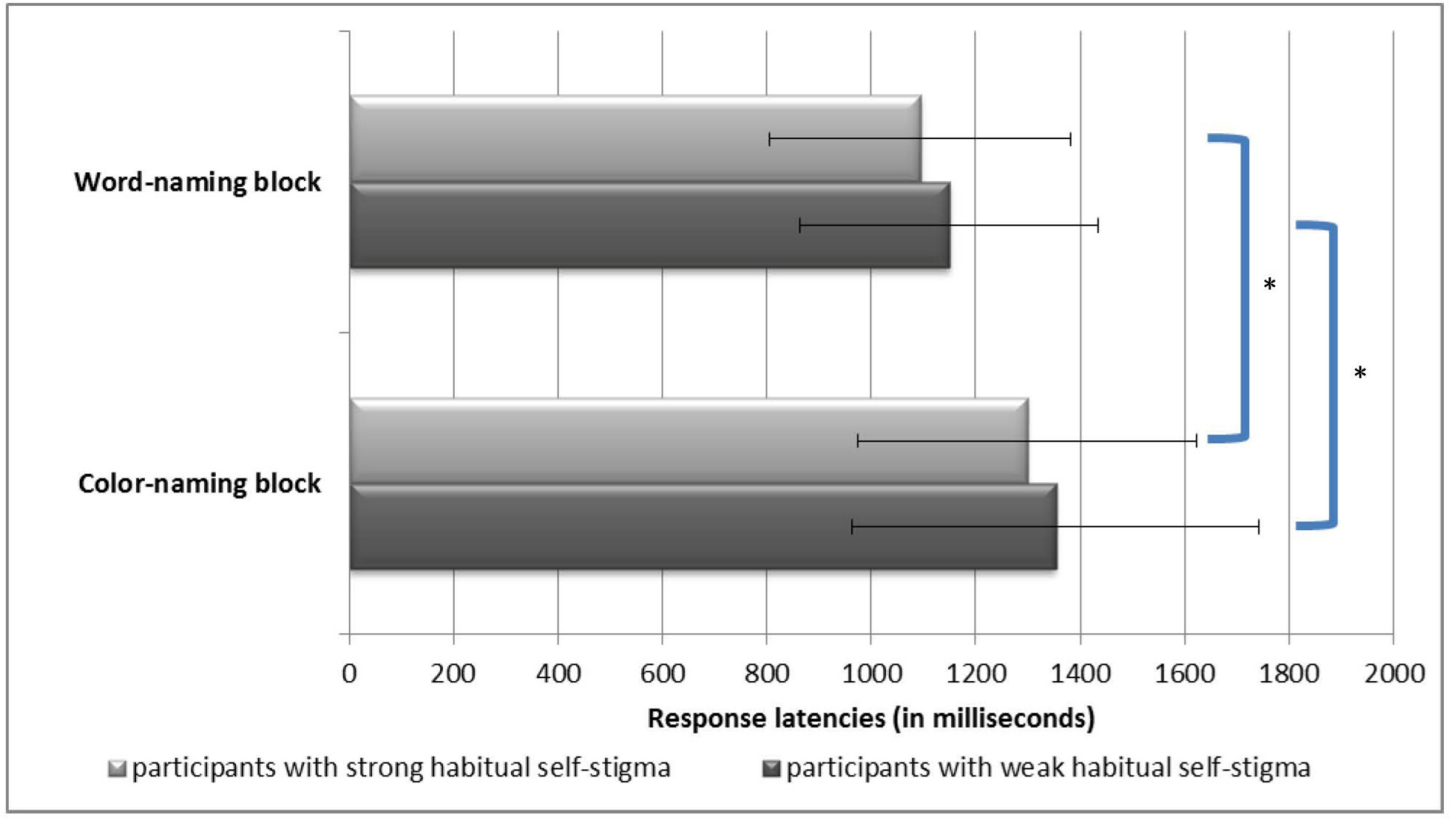
Participants’ performance on the cognitive Stroop task. Mean response latencies in the word-naming and color-naming blocks. An asterisk indicates a significant difference.

A repeated measures ANOVA for the latency scores showed that there was a significant main effect of “Condition” (*F* = 31.20, *p* < 0.001); however, the main effect of “Group” (*F* = 0.81, *p* = 0.37) and the interaction effect of “Group” X “Condition” (*F* < 0.001, *p* = 0.99) were not significant (see Fig 2). Specifically, participants had slower reaction times during the color-naming condition compared to the word-naming condition.

According to the selective attention scores calculated for the cognitive Stroop task, a cognitive Stroop interference effect was present in both the strong (mean = 204.85 ms, standard deviation = 314.10 ms) and weak habit groups (mean = 203.72 ms, standard deviation = 352.53 ms). An independent samples *t*-test showed that there was no significant group difference in the magnitude of the interference effect (*t* = 0.02, *p* = 0.99).

## Discussion

The present study found that people with strong habitual self-stigma were faster to color-name stigma-related words compared to non-affective words, whereas those with weak habitual self-stigma showed no difference in the speed of response to different stimuli. More rigorous analyses using latency difference scores further supported these findings, showing that only the strong habit group exhibited a significant emotional Stroop facilitation effect of stigma-related stimuli. Moreover, such attentional bias was not attributable to the general selective attention competencies in the participants, as the cognitive Stroop effect was present in the strong habit group to the same extent as in the weak habit group. Thus, our findings point to a specific relationship between habitual self-stigma and biased processing of stigma-related material. To the best of our knowledge, this study is the first to demonstrate the role of information-processing bias in self-stigma.

Only the strong habit group, but not the weak habit group, showed emotional Stroop facilitation effects of stigma-related stimuli, suggesting that people with stronger habitual self-stigma may be more able to ignore the semantic meaning of stigma-related words and focus on the color-naming task. These findings are contrary to our hypothesis, but consistent with previous observations of emotional Stroop facilitation for mood-congruent stimuli in some people with social phobia [39] and obsessive-compulsive disorder [40,41]. The present findings, particularly the different reactions of the two participant groups to stigma-related stimuli, may be explained by previous observations of greater experiential avoidance in people with stronger habitual self-stigma [19]. Experiential avoidance refers to the excessive negative evaluation of undesirable thoughts, along with an unwillingness to experience these thoughts, and deliberate efforts to avoid or suppress them [42]. However, the paradox of experiential avoidance is that attempts to avoid or suppress a thought can cause a rebound increase in the frequency of the thought, leading to prolonged preoccupation with it [43]. This avoidant tendency may thus increase the vulnerability of people with mental illness to repetitive and eventually habitual self-stigma [19]. More importantly, if it is true that experiential avoidance may serve to inhibit the processing of stigma-related material, this should also make individuals respond faster to stigma-related words in the emotional Stroop task, such that those threatening words could be removed rapidly from the computer screen as well as the perceptual field. Future research should use more sophisticated experimental paradigms (e.g., the dot-probe task [44,45]) and measurement techniques (e.g., the eye-tracking technology [46]) to directly examine attentional avoidance of stigma-related material in people with mental illness.

Interestingly, on a trend level, the strong habit group exhibited emotional Stroop facilitation for affective stimuli, whereas the weak habit group showed emotional Stroop interference effects. The interference effects are consistent with previous findings of a tendency for affective stimuli to draw more attention than non-affective stimuli [47]. On the other hand, the facilitation effects may be explained by the emotion context insensitivity hypothesis [48], which posits that individuals experiencing a sad mood (e.g., people with strong habitual self-stigma) tend to show diminished emotional reactivity to both positive and negative stimuli. Specifically, these individuals may have a reduced drive to keep engaging with positive or rewarding features of the environment. They may also be less emotionally aroused by negative or aversive situations. Consequently, in the emotional Stroop task, people with strong habitual self-stigma might be more able to divert their attention from the distracting emotional characteristics of affective words. They might also be quicker in re-orienting their attention to the task-relevant feature of the words, resulting in faster response latencies.

The significant relationship between the STAR and the emotional Stroop effect of stigma-related stimuli is important, as this provides solid evidence on the sensitivity of the STAR to automatic cognitive processes (e.g., attentional bias) that are stigma-related. In contrast, the content-oriented measure of self-stigma was not significantly related to the latency measures, providing compelling support for the discriminant validity of the two self-stigma measures. Consistent with many [49–52] but not all [53–54] past studies, we failed to demonstrate a significant relationship between depression and attentional bias for negatively valenced material (i.e., stigma-related words). The inconsistent findings may in part be due to the varying diagnostic groups and affective stimuli investigated in different studies. However, on the upside, as neither stereotype self-concurrence nor depression was significantly related to any of the latency measures, the effects of these potential confounding factors could not explain our findings.

A subsidiary finding was that people with stronger habitual self-stigma exhibited greater levels of stereotype self-concurrence and depression. Stereotype self-concurrence was positively related to habitual self-stigma, likely because individuals with greater stereotype self-concurrence have greater anticipation of personal failure in their daily lives [55], which could, in turn, make their self-stigmatizing cognitions highly salient and more frequently on their minds [17]. On the other hand, the presence of more depressive symptoms in the strong habit group, as found in our study, suggests that habitual self-stigma is associated with poorer mental health outcomes for people with mental illness. This finding, together with other evidence on the adverse effects of habitual self-stigma on self-esteem, life satisfaction, and personal recovery [18,24], suggests that efforts to reduce habitual self-stigma are important and worthy, as the reduction of habitual self-stigma may improve people’s psychological well-being and overall quality of life. In the future, psychological services provided for people with mental illness should consider targeting the frequency and automaticity of self-stigma. In particular, future anti-self-stigma interventions might want to facilitate people to extend their self-definition beyond their patient or minority status, thereby thinking about their stigmatized condition less often (i.e., reducing repetition) [16]. Interventions might also want to address automatic self-stigma by using mindfulness-based approaches [56]. By cultivating mindfulness, people can learn how to observe thought patterns, thereby enabling awareness and monitoring of automatic self-stigmatization [22].

Several limitations should be addressed before drawing any conclusions. First, the present study is cross-sectional in nature, thereby preventing the conclusion of cause-and-effect relationships among the variables. Indeed, prospective longitudinal studies of self-stigma in people with mental illness [10] have been scarce despite the invaluable information they would contribute to the field. Second, by using the extreme groups approach as the sampling method, this study examined the role of attentional bias in habitual self-stigma only among extreme scorers on the STAR. Thus, the present findings are tentative and further evidence is needed from replications of our results based on analyses of full-range, continuous data. Finally, the present study focuses on one specific measure of attentional bias. Thus, our results can only be applicable to the specific components of information-processing biases measured by the emotional Stroop task, and the potential role of other cognitive biases (e.g., memory bias and interpretation bias) in habitual self-stigma is yet to be explored.

## Concluding Remarks

Notwithstanding the above limitations, the theoretical contributions of the present study are evident. Specifically, this study is the first to conceptualize the contributory role of information-processing biases in self-stigma among people with mental illness. Our findings suggest that further examination of the role and the nature of attentional bias in habitual self-stigma is an important avenue for future research. In particular, there is a critical need to adopt a wider range of experimental paradigms (e.g., the dot-probe task) and measurement techniques (e.g., the eye-tracking technology) to study stigma-related attentional bias. With a more comprehensive understanding of the cognitive roots of habitual self-stigma, we can move closer to changing this maladaptive mental habit that bombards many people with mental illness each day.
